# 

**Published:** 1800-07

**Authors:** 


### THE MEDICAL AND PHYSICAL JOURNAL,

CONTAINING THE

EARLIEST INFORMATION ON SUBJECTS

OF

MEDICINE, SURGERY, PHARMACY, CHEMISTRY,

AND

NATURAL HISTORY;

AND A

CRITICAL ANALYSIS OF ALL NEW BOOKS

IN THOSE

DEPARTMENTS OF LITERATURE.

CONDUCTED BY

T. BRADLEY, M.D.

Member of the Royal College of Phyſicians, London; Phyſician to the Weſtminſter Hoſpital, and to the Aſylum for Female Orphans; Lecturer on the Theory and Practice of Medicine, &c.

BY

R. BATTY, M.D.

Licentiate in Midwifery, of the Royal College of Phyſicians, London; of the Britiſh Lying-In-Hoſpital; Phyſician to the Infant Aſylum; Fellow of the Linnean Society; and Lecturer on Midwifery.

AND BY

A. A. NOEHDEN, M.D.

Vice-Secretary of the Phyſical Society of Gottingen; Correſponding Member of the Linnean Society, London, and of the Medical Society at Paris.

VOL. IV.

FROM JUNE TO DECEMBER, 1800.

Washington, D.C.

*LONDON:*

Printed for R. PHILLIPS, No. 71, ST. PAUL'S CHURCH-YARD; and ſold by all Bookſellers in the United Kingdoms.

*Price Twelve Shillings in Boards.*

[WILLIAM THORNE, Printer, Red Lyon Court, Fleet Street.]

[ Entered at Stationers Hall.

### ADVERTISEMENT.

*It is unnecessary for us to enumerate the advantages the World derives from the different branches of Medicine. The state of health enjoyed by most human beings, at some period or other of their lives, impresses this fact too forcibly on their minds to render it a subject of doubt. To most of the readers of the Medical and Physical Journal, who are probably members of the Faculty, still less need be said on this subject, every practitioner being thoroughly convinced that much real benefit is daily arising from those who lose no opportunity of acquiring information, and immediately applying the skill and discoveries of their brethren to the best advantage.*

*We trust that our endeavours in so widely diffusing the aggregate labours of individuals have contributed materially to this desirable end; indeed, if we may be allowed to judge from the flattering [illegible]stimonies we daily receive from our Correspondents, and from [illegible] circulation which the Medical and Physical Journal now universally has through the Navy and Army, we are confident that the benefits accruing to our brave champions and defenders must be inestimable, and the improvement in Naval and Military Physic and Surgery incalculable.*

*In retracing the various subjects in this Volume, our readers will agree with us, that very valuable additions have been made to our knowledge of the Cow-pock; that its discussion in our Journal has extended the benefits of this discovery to the world; and the minute inquiry into some occasional irregularities attending it, has led to a certain mode of practice, securing the happy effects mankind are taught to expect from it.*

*On the practice of Midwifery, and on the management of the Placenta, we are persuaded that considerable benefit must result from the valuable communications and practical remarks we have inserted on this important branch of medicine.*

*We are glad to observe that Physicians are now adopting the affusion of cold water in the hot stage of fever, a practice introduced by the ingenious and learned Dr. Currie, who is entitled to the thanks of the Faculty for a powerful remedy, and precise rules for its application; on this head we have conveyed some additional*

*ditional testimonies to its safety and efficacy.—On the subject of Pulmonary Consumption, and the exhibition of Digitalis, we are led to hope for cures in an almost hopeless class of maladies; and although we may be occasionally disappointed in our expectations of its certainly fulfilling our wishes, it is undoubtedly a powerful agent; and when more experiments and facts shall be collected, and more precision is observed in its preparation and exhibition, we are convinced the profession, as well as the community, will not only reap the fruits of it, but confess their obligation to those who have brought it into notice.*

*We also trust that the public will eventually derive benefit from our communications on Cancer, and the discussion it has given rise to.—If, as yet, many new facts are not added to our stock; if we are still necessitated to have recourse to palliatives, and the feeble practice of prescribing solely to the most urgent symptoms; if we have not yet actually established a just theory, or discovered an efficacious remedy for this dreadful disease,—we are glad to find that bold assertions are received with proper caution; that statements of cases are carefully scrutinized and cross-examined; that men of science are eager to unveil mystery, and afford the world and suffering humanity, a fairer opportunity of estimating what degree of confidence may be placed on superior pretensions, or on partial and ambiguous representations of facts. However, if it be true that civil convulsions call forth unexampled energies and unexpected abilities, and if the blaze of truth is frequently elicited from the collision of jarring opinions, we have a right to hope that a full and severe investigation of the pathology and supposed remedies of this horrible disease will at last reward us for the search.*

*The state of science at the close of the eighteenth century gives us a good claim to our expectations. Deep anatomical and physiological knowledge are not now confined to one or two Professors at our Universities;* EVERY PRACTITIONER *must lay a sound foundation of the principles of Medicine, to stand in fair competition with his cotemporaries; and we sincerely rejoice that the very extensive circulation of the Medical and Physical Journal so evidently facilitates the communication of information, and tends so rapidly and effectually to the improvement of the healing art.*

LONDON, DEC. 13, 1800.

### NEW MEDICAL PUBLICATIONS IN JUNE.

The Edinburgh Practice of Phyſic and Surgery, preceded by an Abſtract of the Theory of Medicine, and the Noſology of Dr. Cullen, and including upwards of 500 authentic Formulæ from the Books of the London Hoſpitals, and from the Lectures and Writings of the moſt eminent public Teachers; with Plates, 8vo. 14s. boards. Kearſley.

Obſervations on Mr. Simmons's Detection, &c. With a Defence of the Ceſarean Operation, an Examination of Dr. Oſborn's Opinions relative to Embryulcia, and an Account of the Method of Delivering by Embryotomy; by JOHN HULL, M. D. 8vo. 9s. boards. Bickerſtaff.

Obſervations on the Gout and Rheumatiſm; to which are annexed Phænomena Phyſiologica, iſſuing in the Cure of thoſe Diſeaſes; by William Peter Whyte; 2s. 6d. boards. Rivingtons.

Memorials on the Medical Department of Naval Service, tranſmitted to the Lords of the Admiralty; to which is annexed, an Addreſs to Parliament on the Expediency of amending the Laws relative to the Exportation of Corn; by WILLIAM RENWICK, Surgeon in the Royal Navy, 1s. Longman.

---

### TO CORRESPONDENTS.

*We are much obliged to our Correspondent at Newcastle, for the zeal he has discovered in the promotion of an important improvement in the practice of medicine; but we beg leave to assure him, that such paragraphs as those he has read in the Newcastle Advertiser, can never "prevent the adoption of the Vaccine Inoculation" in any part of the kingdom, while it continues to be supported and encouraged by the most respectable practitioners. A paragraph or letter, very similar to the one he sent us a copy of, appeared a few weeks ago in a London Paper, but the Faculty here treated it with the silent contempt it merited; being persuaded that the declamatory effusions of such writers, when opposed to the opinions of Jenner, Woodville, Pearson, Denman, Saunders, Cline, Keate, Ring, Knight, Abernethy, and many others equally respectable, can have no weight with a discerning public. If, however, our Correspondent really believes that such paragraphs, with such signatures, or without a name, can possibly operate to the discouragement of the New Inoculation, in parts distant from the metropolis, we deem it will be sufficient for him, or any other of our Correspondents, to refer their sceptical patients to the exalted and respectable names which appear in the first pages of this Number.*

*A Pharmaceutical Correspondent concludes, from the Case given at page* 535 *of the Third Volume, that corrosive sublimate is either much cheaper in the neighbourhood of Ketley than in London, or that the article sold there was adulterated.*

*Communications are received from Dr.* Alderſon *and Mr.* Watt.

